# Supplementary material for: The Extracellular Matrix Proteins Tenascin-C and Tenascin-R Retard Oligodendrocyte Precursor Maturation and Myelin Regeneration in a Cuprizone-Induced Long-Term Demyelination Animal Model
Source: Cells. 2022 May 28;11(11):1773. doi: 10.3390/cells11111773 (PMC9179356; doi:10.3390/cells11111773)
Supplement: Supplementary file 1 [file cells-11-01773-s001.zip › cells-1686694-supplementary.pdf]

Supplement to "The Extracellular Matrix Proteins Tenascin-C and Tenascin-R Retard Oligodendrocyte Precursor Maturation and Myelin Regeneration in a Cuprizone-Induced Long-Term Demyelination Animal Model" by Juliane Bauch and Andreas Faissner.

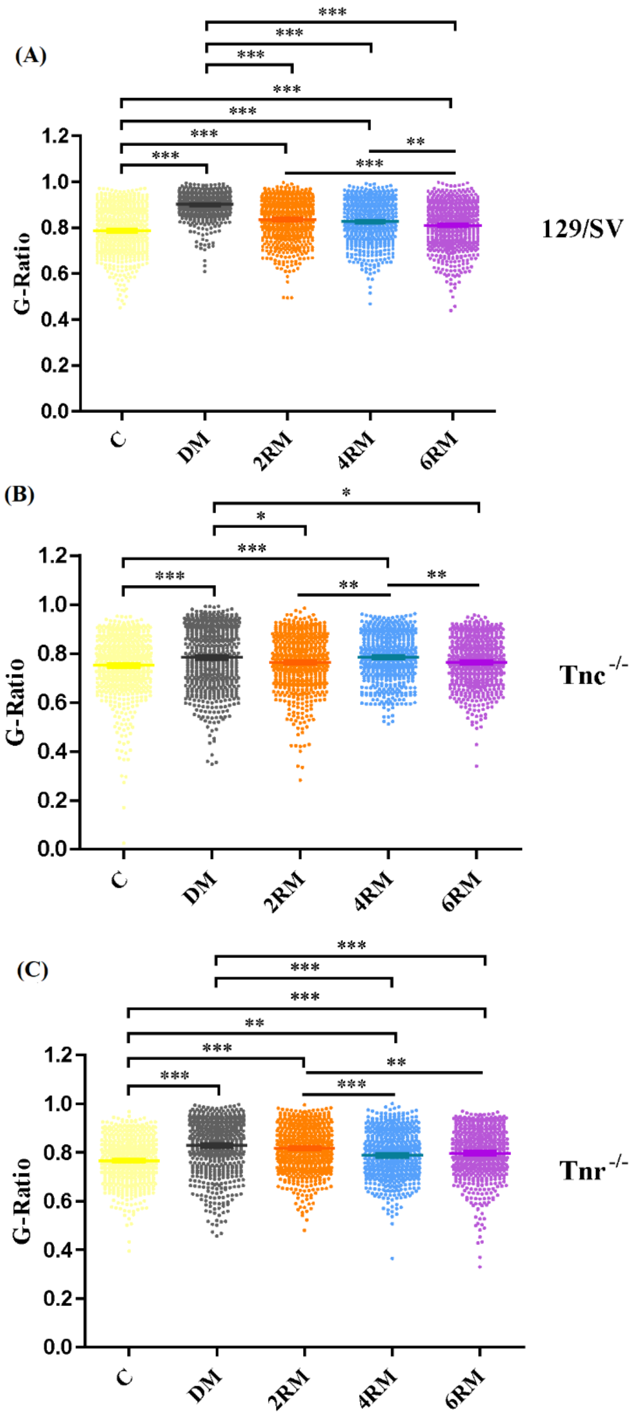

Suppl. Figure S1: G-ratios under control, de- and remyelination conditions. Electron microscopy analysis confirmed successful cuprizone induced demyelination in each genotype. 8 until 10-week-old male mice from different genotypes (129/SV as wildtype, *Tnc*$^{-/-}$ and *Tnr*$^{-/-}$) received either a normal diet as untreated control or a 0.2 % cuprizone diet to induce demyelination (A-C). After 10 weeks mice were either perfused or received a normal diet for 2, 4 or 6 further weeks to allow for remyelination after withdrawal. The results confirmed the cuprizone induced demyelination (A-C). g-ratios of demyelinated

tissues were significantly higher than in untreated control condition in each genotype. Successful remyelination occured in each condition and is represented by decreasing g-ratios. For statistical analysis, the unpaired two-tailed student´s test ($p \leq 0.05$ *, $p \leq 0.01$ **, $p \leq 0.001$ ***) was used. Axon diameter seemed smaller in *Tnc*$^{-/-}$ and *Tnr*$^{-/-}$ mice. Statistical analysis was carried out by using the ANOVA and Tukey's multiple comparison test (N=3, n≤594).

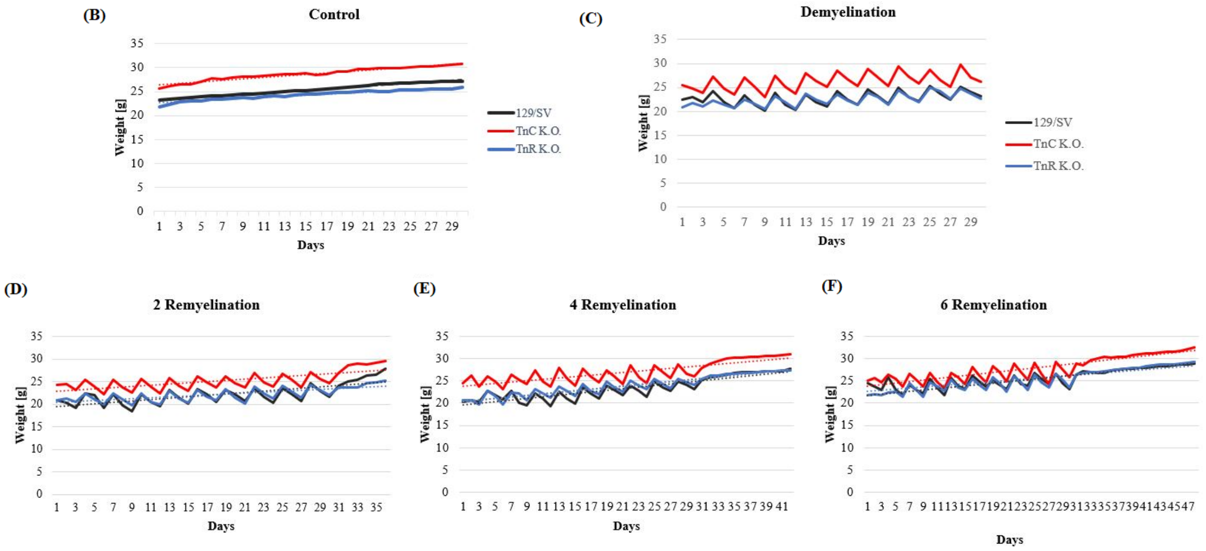

Suppl. Figure S2: Body weights under control, de- and remyelination conditions. Weight documentation during, without and after cuprizone treatment in the different genotypes (SV/129, *Tnc*$^{-/-}$ and *Tnr*$^{-/-}$). Weight changes of the mice were documented three times a week. During control condition weight gained steadily over time in each genotype. During demyelination the weight of the mice decreased over time and was indicative of successful demyelination as a result of cuprizone treatment. Once a week cuprizone treated mice received a normal diet to minimize the severity of intoxication and therefore interim peaks were detectable. With ongoing remyelination time the weight of the mice increased in each genotype, which is an indicator for successful remyelination.
